# Supplementary material for: Single-cell analysis reveals the stromal dynamics and tumor-specific characteristics in the microenvironment of ovarian cancer
Source: Commun Biol. 2024 Jan 5;7:20. doi: 10.1038/s42003-023-05733-x (PMC10770164; doi:10.1038/s42003-023-05733-x)
Supplement: Supplementary file 2 — Supplementary Information [file 42003_2023_5733_MOESM2_ESM.pdf]

Supplementary Materials for

Single-cell analysis reveals the stromal dynamics and tumor-specific characteristics in the microenvironment of ovarian cancer

Linan Zhang, Sandra Cascio, John W Mellors,
Ronald J Buckanovich, Hatice Ulku Osmanbeyoglu

*Corresponding author. Email: osmanbeyogluhu@pitt.edu

This PDF file includes:

Figures S1 to S11
Tables S1 to S5
References (1 to 10)

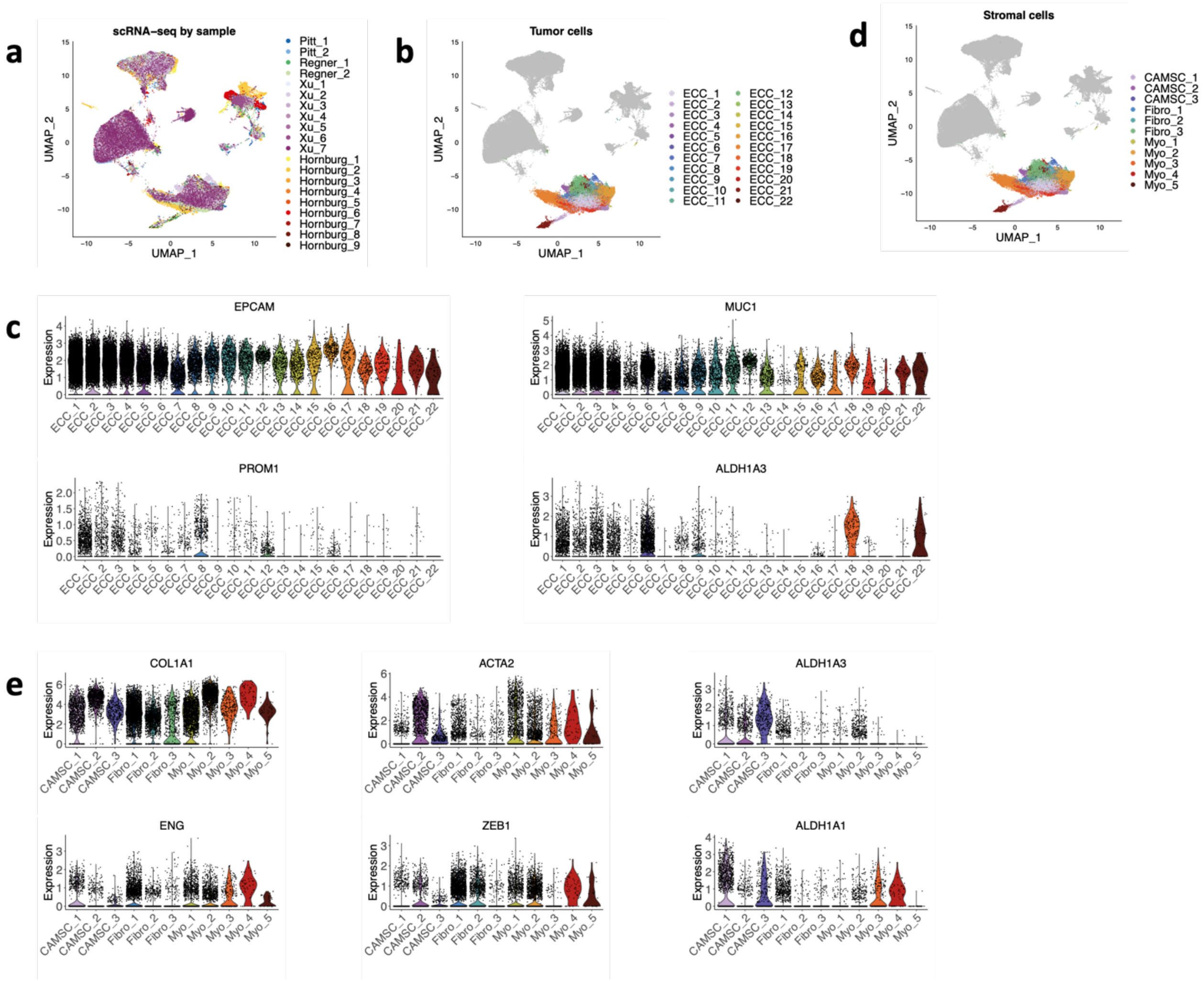

Figure S1. Extended view of HGSOC samples. a Uniform manifold approximation and projection (UMAP) clustering of the integrated scRNA-seq data, colored by sample. Each dot represents a single cell. **b** UMAP plot of subclusters of tumor cells. **c** Violin plots showing the expression of *EPCAM*, *MUC1*, *PROM1*, and *ALDH1A3* in tumor cells by subcluster. **d** UMAP plot of subclusters of stromal cells. **e** Violin plots showing the expression of *COL1A1*, *ACTA2*, *ALDH1A3*, *ENG*, *ZEB1*, and *ALDH1A1* in stromal cells by subcluster.

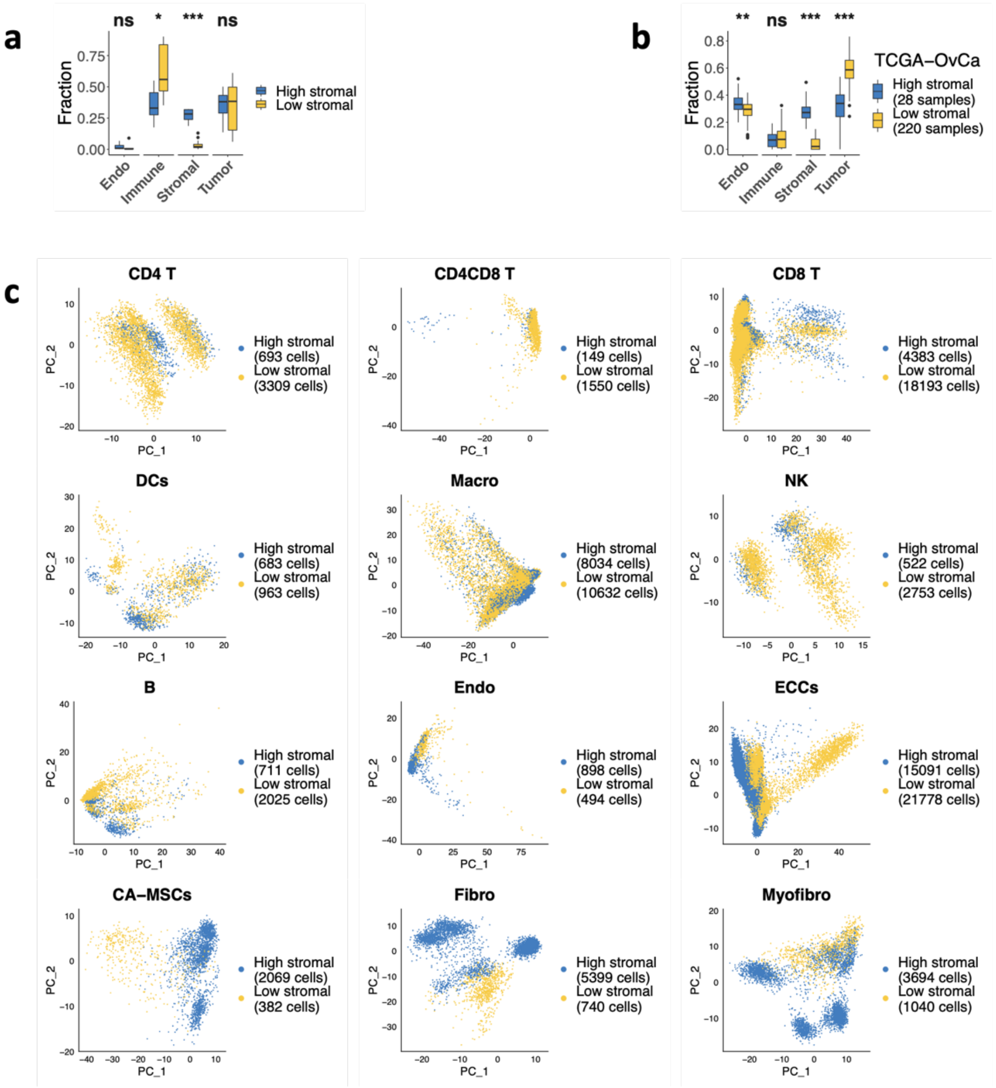

Figure S2. Different landscapes between high- and low-stromal tumors. a Box plot showing the fractions of endothelial, immune, stromal, and tumor cells in each sample, colored by tumor group (high-/low-stromal groups). The *p*-values are computed from the two-sided Wilcoxon signed-rank test between the two groups for each cell type. Statistical significance is coded by the following symbols: . *p*-value<0.1, * *p*-value < 0.05, ** *p*-value < 0.01, and *** *p*-value < 0.001. **b** Principal component analysis (PCA) and scatter plots along the top 2 PCs for each cell type. Each dot represents a single cell. **c** Box plot showing the inferred cell type fractions in each sample of the TCGA-OvCa dataset, colored by tumor group.

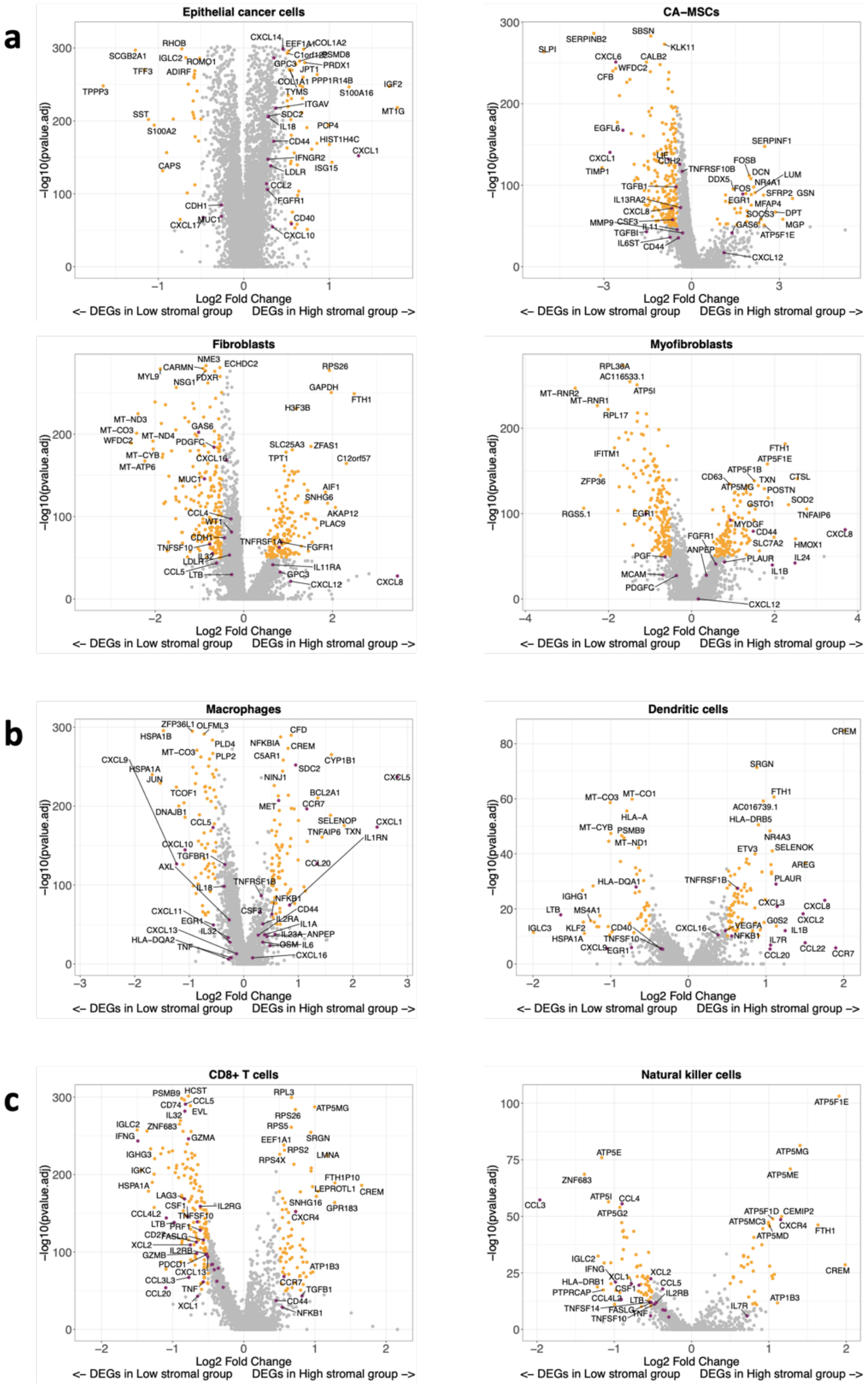

Figure S3. Differentially expressed genes. a Volcano plots showing differentially expressed genes (DEGs) in epithelial cancer cells, CA-MSCs, fibroblasts, and myofibroblasts between high- and low-stromal groups. DEGs (colored in orange) were identified using the Seurat's *FindMarkers* function. In particular, differentially expressed cytokines and surface proteins are colored in purple. Genes with positive log2 fold change are upregulated in the high-stromal group, and vise versa. **b** Volcano plots showing DEGs in macrophages and dendritic cells. **c** Volcano plots showing DEGs in $CD8^{+}$ T cells and natural killer cells.

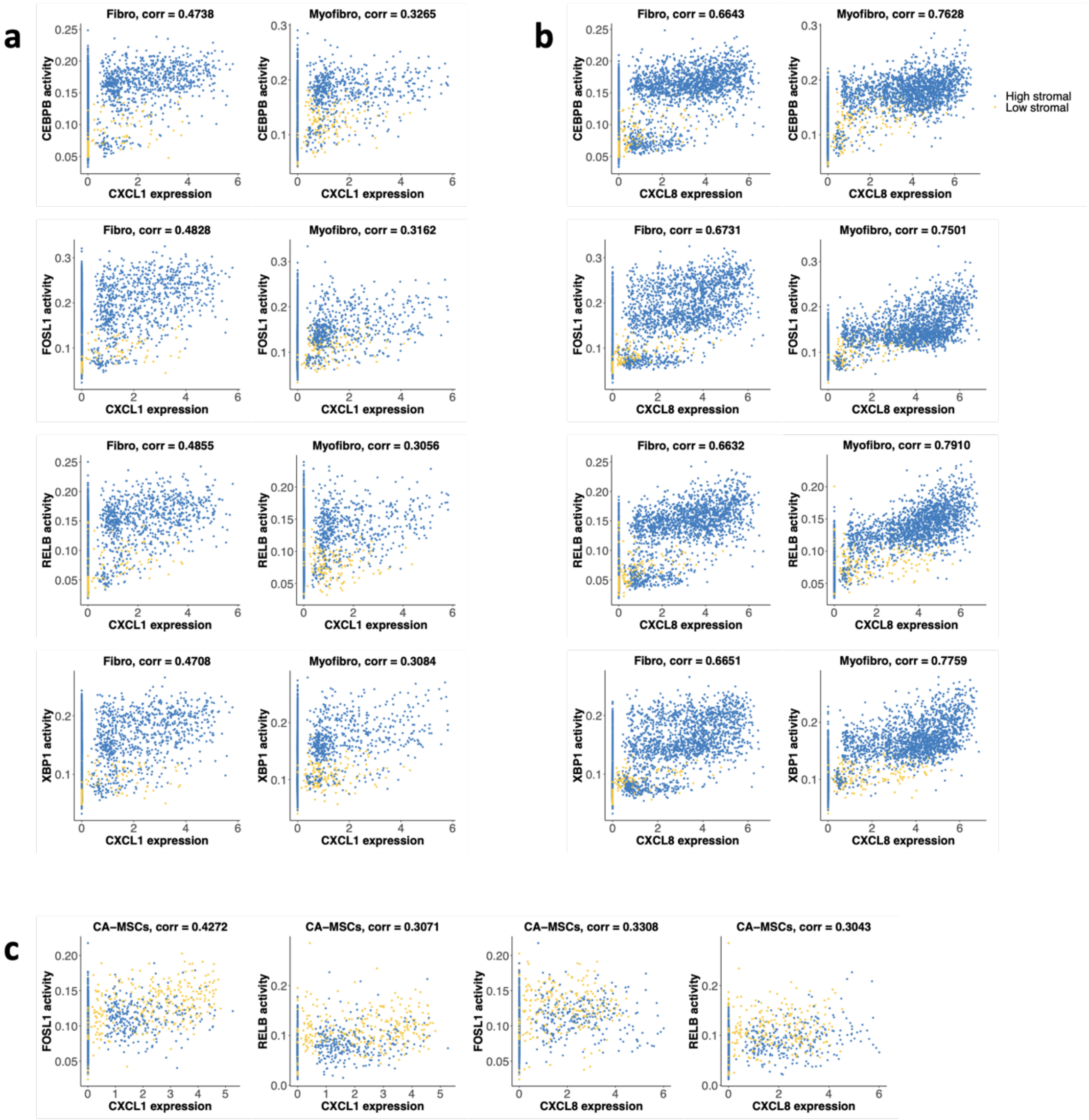

Figure S4. Highly correlated gene-TF pairs in stromal population. a Scatter plots of selected highly correlated *CXCL1*-TF pairs and **b** *CXCL8*-TF for fibroblasts and myofibroblasts. Each dot represents a cell, colored by the tumor group. **c** Scatter plots of selected highly correlated *CXCL1*-TF and *CXCL8*-TF pairs for CA-MSCs.

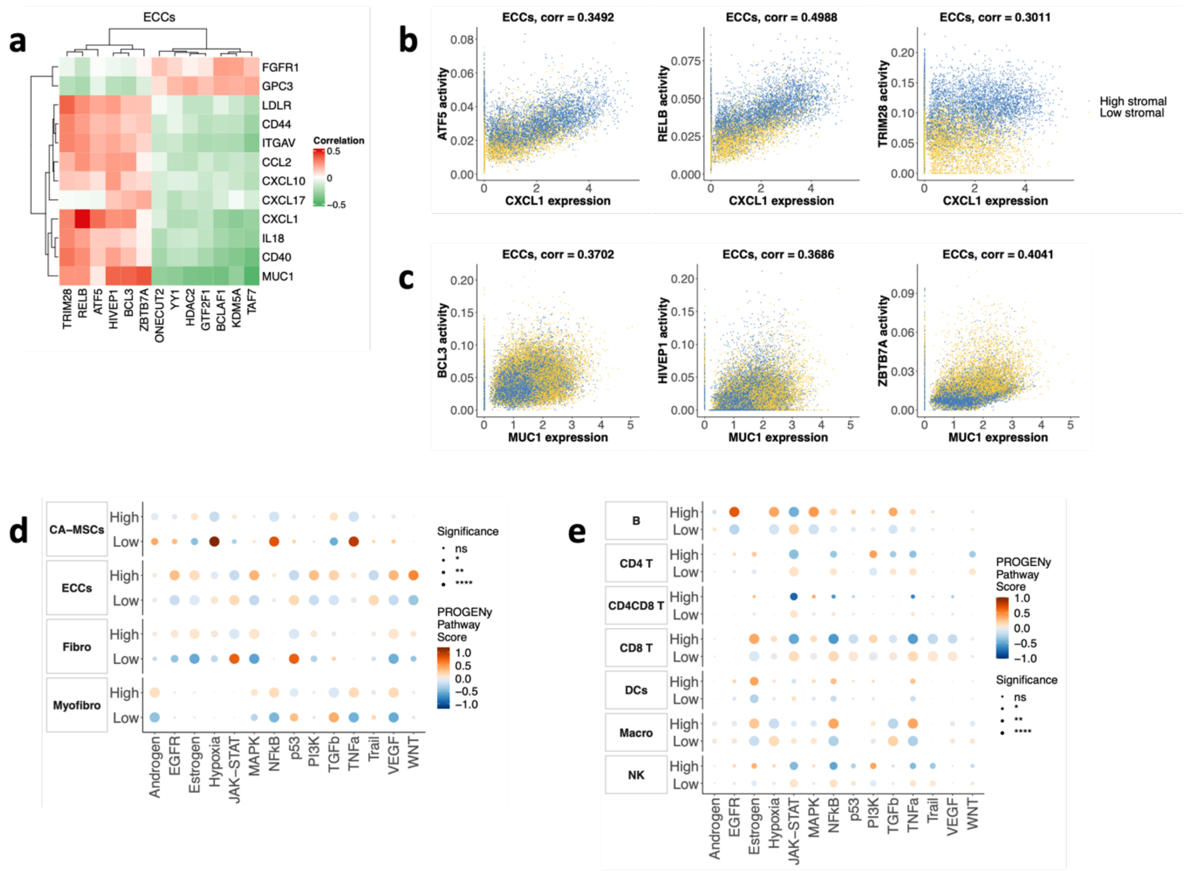

Figure S5. Transcription factor activity analysis and pathway analysis. a Heatmap revealing correlations between inferred TF activities (columns) and surface protein (SP)/cytokine expression (rows) in epithelial cancer cells (ECCs). For clarity, highly correlated SPs/cytokines-TF pairs were selected (**Table S4**). **b** Scatter plots of selected highly correlated *CXCL1*-TF pairs and **c** *MUC1*-TF pairs for ECCs. Each dot represents a cell, colored by the tumor group. **d e** PROGENy pathway score by cell type. For each pathway signaled in each cell type, the two-sided Wilcoxon test is applied between the high- and low-stromal groups; the resulting *p*-values are adjusted based on Bonferroni correction using all pathways in the dataset. Adjusted *p*-values are indicated by circle size.

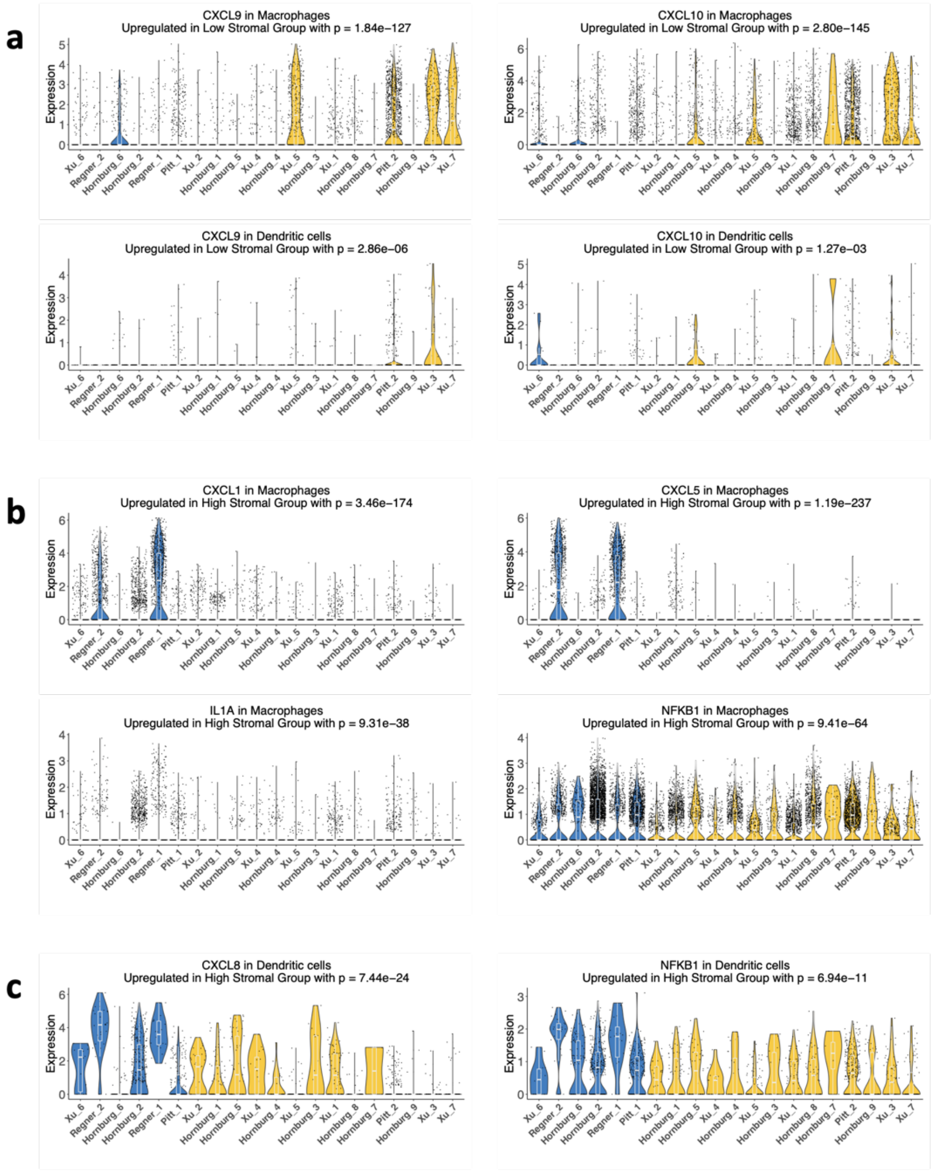

Figure S6. Differentially expressed genes of macrophages and dendritic cells. a Violin plots showing the expression of *CXCL9* and *CXCL10* in macrophages and dendritic cells, **b** the expression of *CXCL1*, *CXCL5*, *IL1A*, and *NFKB1* in macrophages, and **c** the expression of *CXCL8* and *NFKB1*in dendritic cells by sample. Each sample is color-coded by high- (blue) or low- (yellow) stromal group. The *p*-values are computed from the two-sided Wilcoxon tests, adjusted based on Bonferroni correction using all genes in the dataset. Statistical significance is coded by the following symbols: . *p*-value<0.1, * *p*-value < 0.05, ** *p*-value < 0.01, and *** *p*-value < 0.001.

a

b

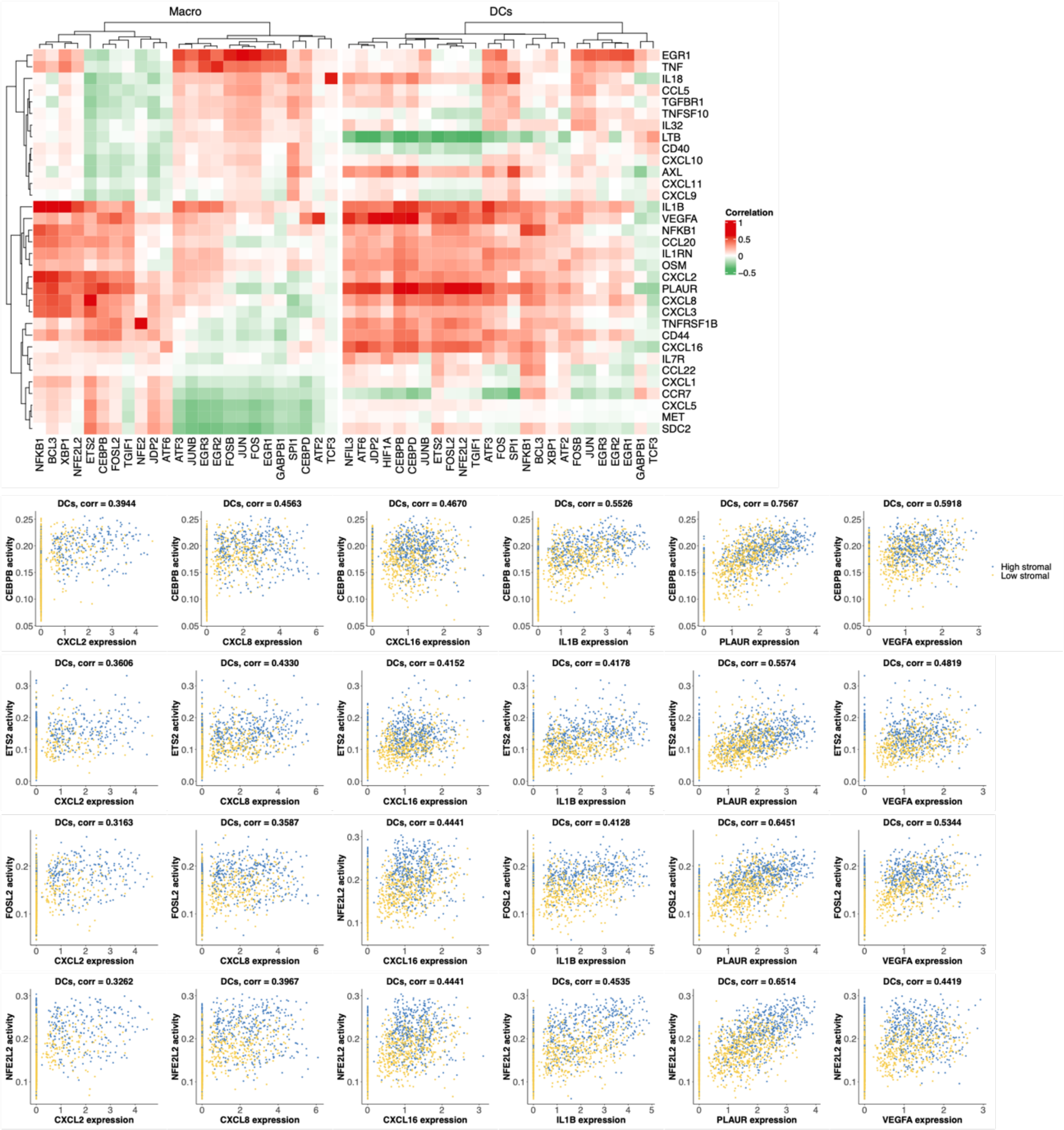
Macro
DCs
EGR1
TNF
IL18
CCL5
TGFBR1
TNFSF10
IL32
LTB
CD40
CXCL10
AXL
CXCL11
CXCL9
IL1B
VEGFA
NFKB1
CCL20
IL1RN
OSM
CXCL2
PLAUR
CXCL8
CXCL3
TNFRSF1B
CD44
CXCL16
IL7R
CCL22
CXCL1
CCR7
CXCL5
MET
SDC2
NFKB1 BCL3 XBP1 NFE2L2 ETS2 CEBPB FOSL2 TGIF1 NFE2 JDP2 ATF6 ATF3 JUNB EGR3 EGR2 FOSB JUN FOS EGR1 GABPB1 SPI1 CEBPD ATF2 TCF3
NFIL3 ATF6 JDP2 HIF1A CEBPB CEBPD JUNB ETS2 FOSL2 NFE2L2 TGIF1 ATF3 FOS SPI1 NFKB1 BCL3 XBP1 ATF2 FOSB JUN EGR3 EGR2 EGR1 GABPB1 TCF3
Correlation
1
0.5
0
−0.5
DCs, corr = 0.3944
DCs, corr = 0.4563
DCs, corr = 0.4670
DCs, corr = 0.5526
DCs, corr = 0.7567
DCs, corr = 0.5918
CEBPB activity
High stromal
Low stromal
DCs, corr = 0.3606
DCs, corr = 0.4330
DCs, corr = 0.4152
DCs, corr = 0.4178
DCs, corr = 0.5574
DCs, corr = 0.4819
ETS2 activity
DCs, corr = 0.3163
DCs, corr = 0.3587
DCs, corr = 0.4441
DCs, corr = 0.4128
DCs, corr = 0.6451
DCs, corr = 0.5344
FOSL2 activity
NFE2L2 activity
DCs, corr = 0.3262
DCs, corr = 0.3967
DCs, corr = 0.4441
DCs, corr = 0.4535
DCs, corr = 0.6514
DCs, corr = 0.4419
NFE2L2 activity
CXCL2 expression
CXCL8 expression
CXCL16 expression
IL1B expression
PLAUR expression
VEGFA expression

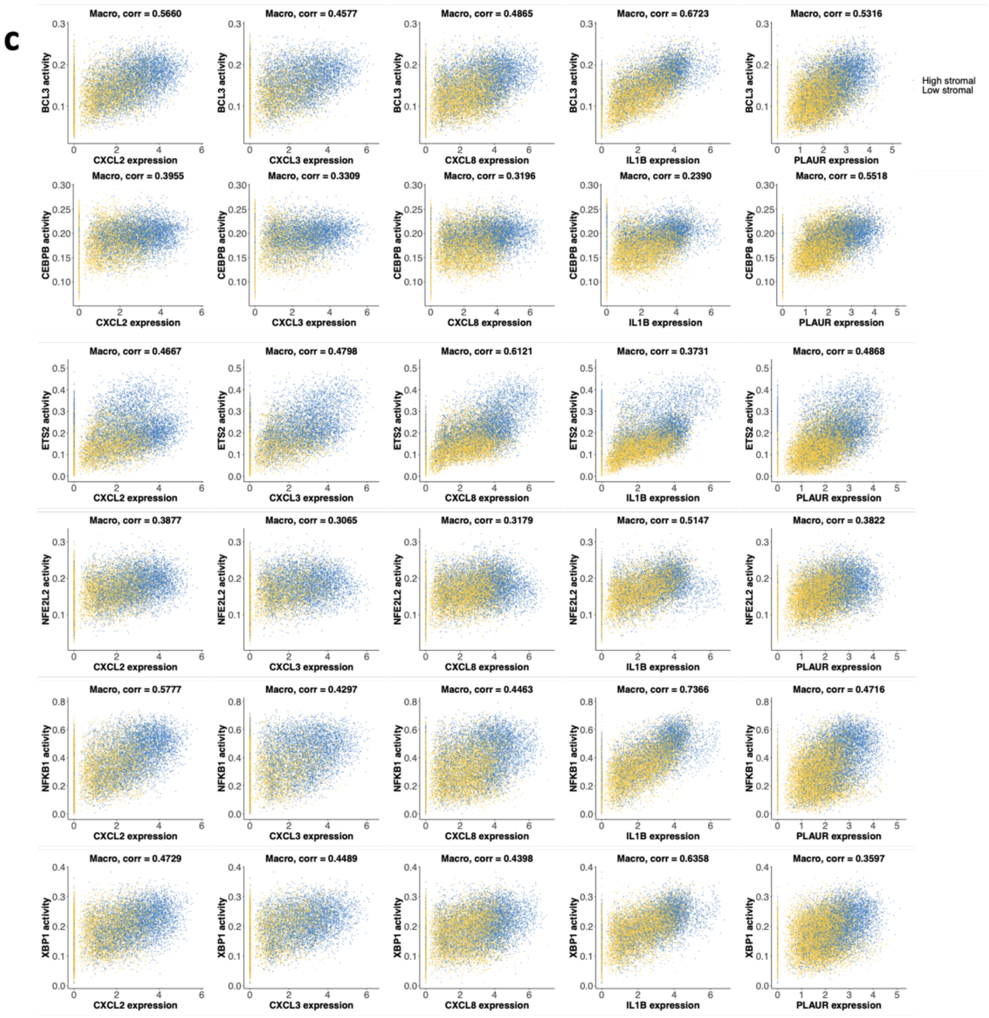

Figure S7. Highly correlated gene-TF pairs in macrophages and dendritic cells. a Heatmap revealing correlations between inferred TF activities (columns) and SP/cytokine expression (rows) in macrophages and dendritic cells (DCs). For clarity, we selected highly correlated SP/cytokine-TF pairs (**Table S4**) and used the union of the selected genes. The correlation coefficients of the top correlated TFs with each SP/cytokine are shown for each cell type. **b c** Scatter plots of selected highly correlated gene-TF pairs for each cell type. Each dot represents a cell, colored by the tumor group.

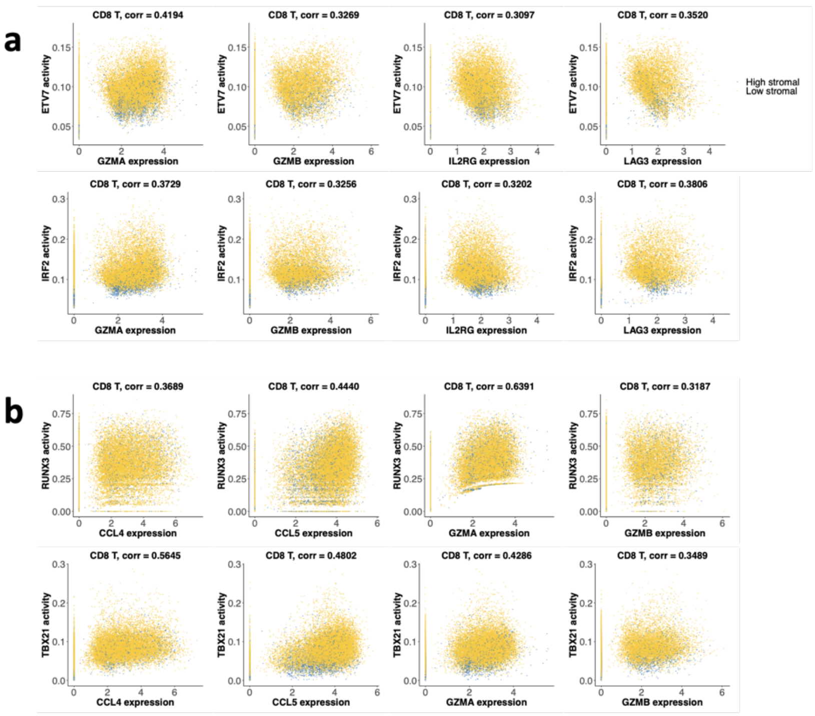
a
CD8 T, corr = 0.4194
CD8 T, corr = 0.3269
CD8 T, corr = 0.3097
CD8 T, corr = 0.3520
ETV7 activity
GZMA expression
GZMB expression
IL2RG expression
LAG3 expression
High stromal
Low stromal
CD8 T, corr = 0.3729
CD8 T, corr = 0.3256
CD8 T, corr = 0.3202
CD8 T, corr = 0.3806
IRF2 activity
GZMA expression
GZMB expression
IL2RG expression
LAG3 expression
b
CD8 T, corr = 0.3689
CD8 T, corr = 0.4440
CD8 T, corr = 0.6391
CD8 T, corr = 0.3187
RUNX3 activity
CCL4 expression
CCL5 expression
GZMA expression
GZMB expression
CD8 T, corr = 0.5645
CD8 T, corr = 0.4802
CD8 T, corr = 0.4286
CD8 T, corr = 0.3489
TBX21 activity
CCL4 expression
CCL5 expression
GZMA expression
GZMB expression

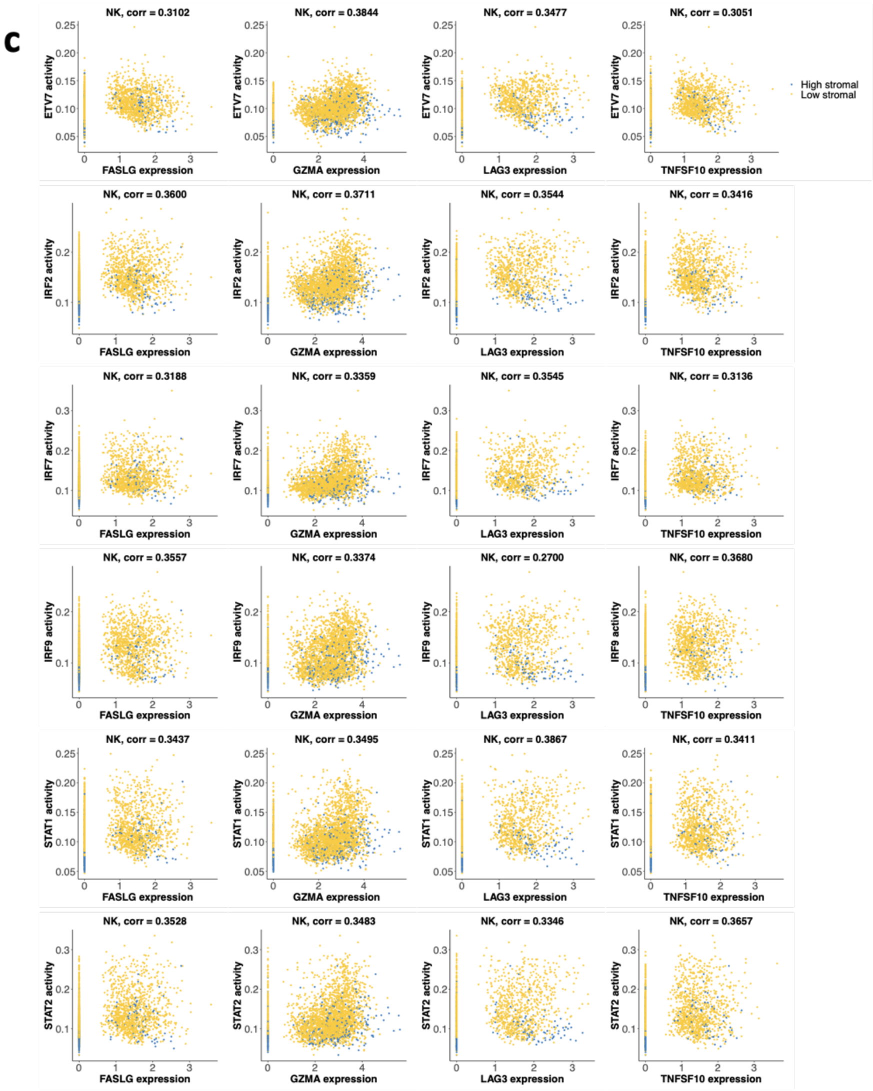

Figure S8. Highly correlated gene-TF pairs in CD8$^+$ T and NK cells. a b Scatter plots of selected highly correlated gene-TF pairs for CD8$^+$ T cells and **c** for natural killer cells (NK). Each dot represents a cell, colored by the tumor group.

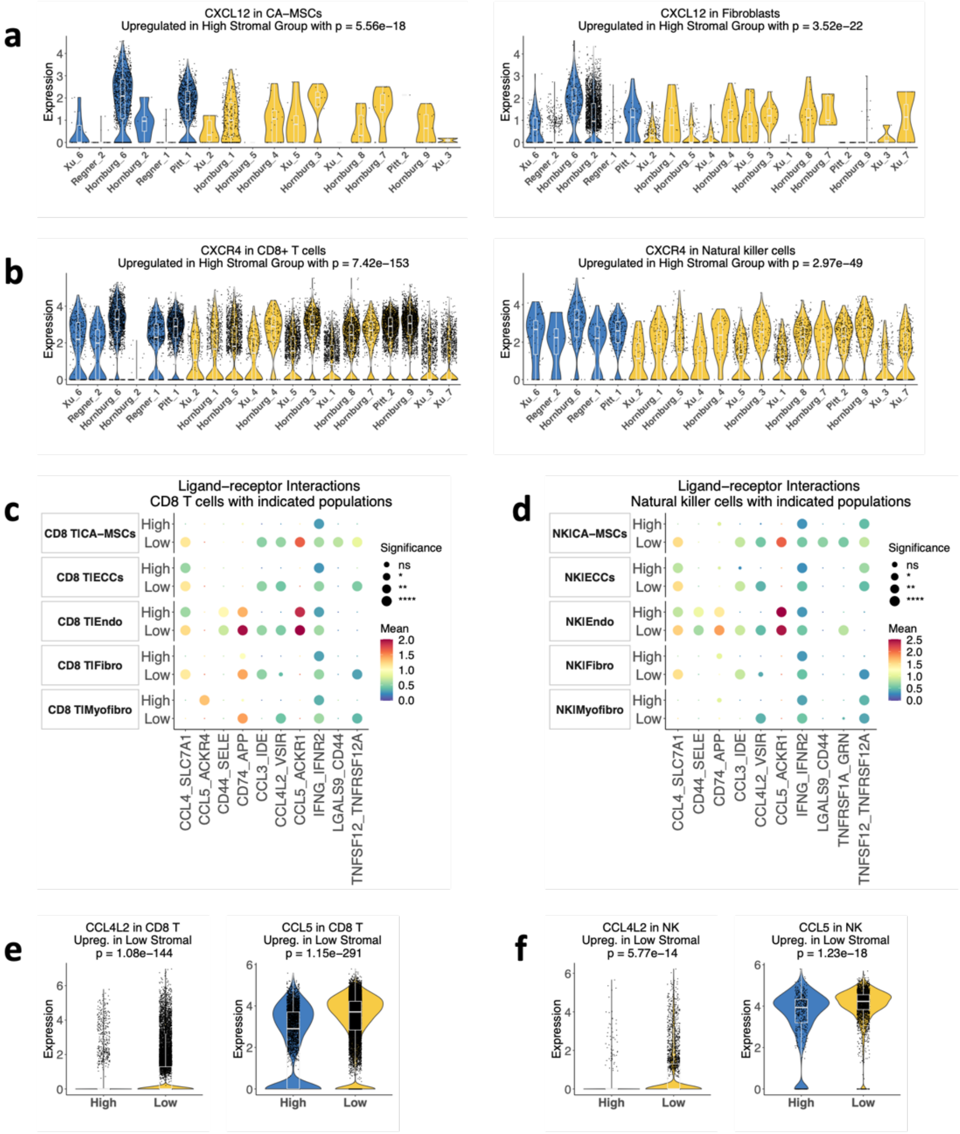

Figure S9. Cell-cell integrations between CA-MSCs/fibroblasts and CD8$^+$ T/NK cells. a Violin plots showing the expression of *CXCL12* in CA-MSCs and fibroblasts by sample Each sample is color-coded by high- (blue) or low- (yellow) stromal group. **b** Violin plots showing the expression of *CXCR4* in CD8$^+$ T and natural killer (NK) cells by sample. **c d** Statistically significant interactions between CD8$^+$ T/NK cells and other cell types using the CellPhoneDB pipeline. Size indicates *p*-values, and color indicates the means of the receptor-ligand pairs between the two tumor groups. **e f** Violin plots showing the expression of *CCL4L2* and *CCL5* in CD8$^+$ T and NK cells by tumor group.

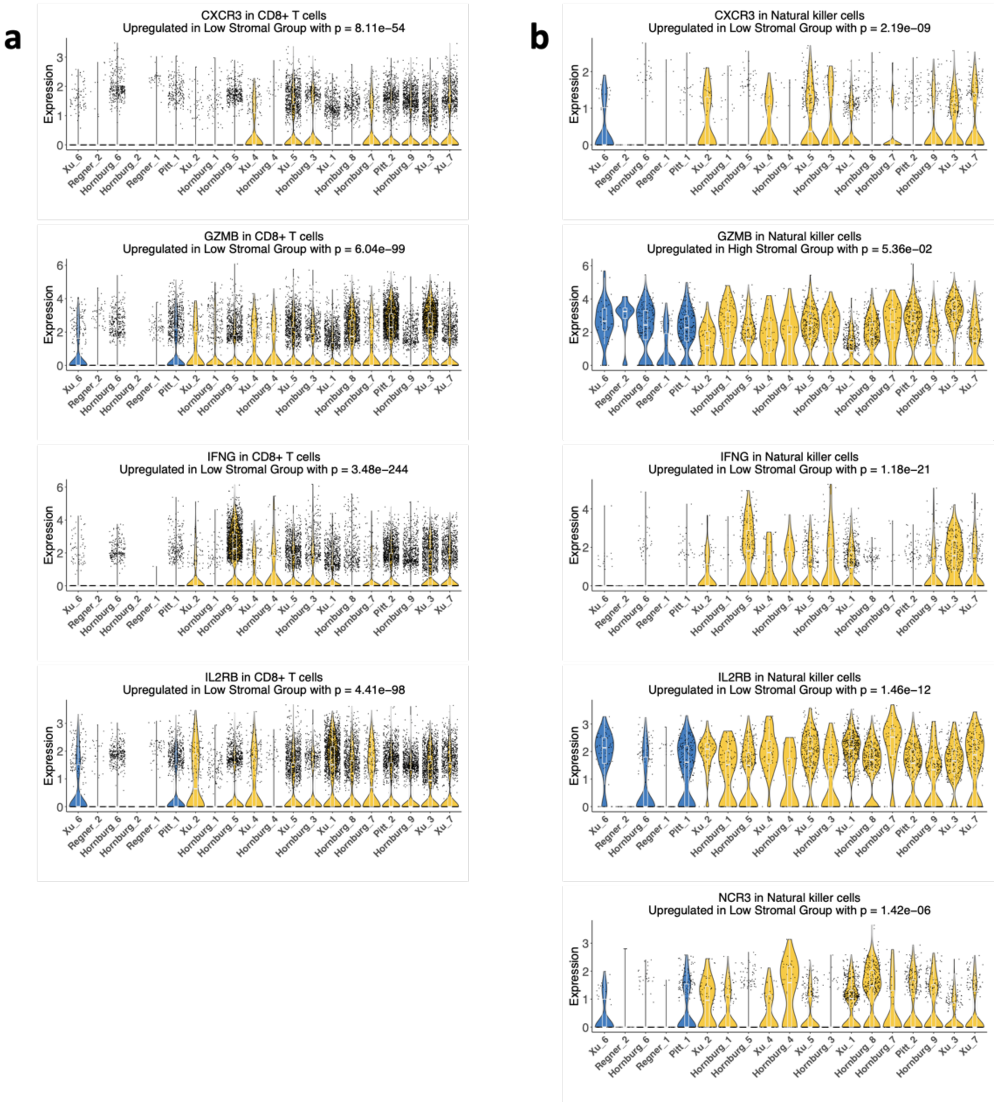

Figure S10. Expression of markers genes associated with activated CD8$^+$ T and natural killer cells. a Violin plots showing the expression of *CXCR3*, *GZMB*, *IFNG*, and *IL2RB* in CD8$^+$ T cells by sample. Each sample is color-coded by high- (blue) or low- (yellow) stromal group. **b** Violin plots showing the expression of *CXCR3*, *GZMB*, *IFNG*, *IL2RB*, and *NCR3* in natural killer cells by sample.

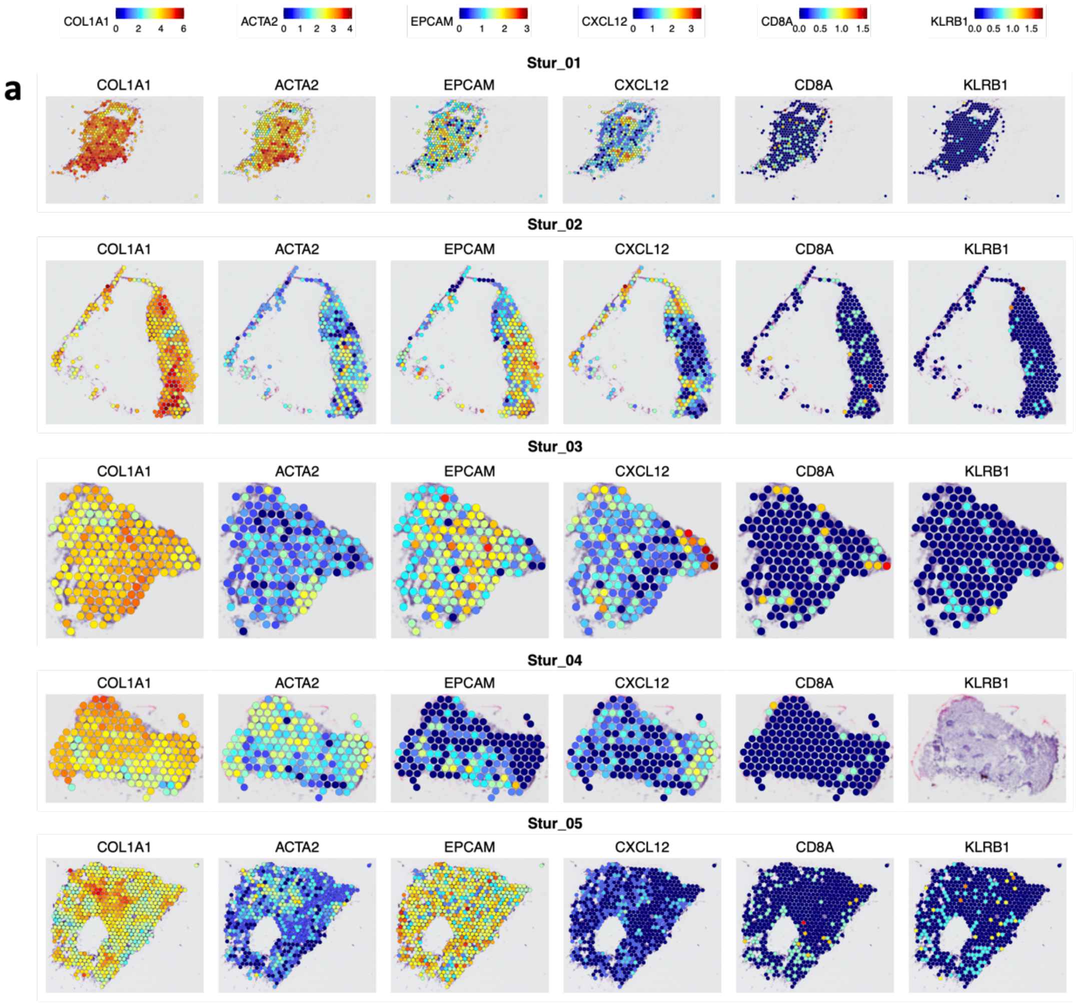
COL1A1 0 2 4 6
ACTA2 0 1 2 3 4
EPCAM 0 1 2 3
CXCL12 0 1 2 3
CD8A 0.0 0.5 1.0 1.5
KLRB1 0.0 0.5 1.0 1.5
a
Stur_01
COL1A1 ACTA2 EPCAM CXCL12 CD8A KLRB1
Stur_02
COL1A1 ACTA2 EPCAM CXCL12 CD8A KLRB1
Stur_03
COL1A1 ACTA2 EPCAM CXCL12 CD8A KLRB1
Stur_04
COL1A1 ACTA2 EPCAM CXCL12 CD8A KLRB1
Stur_05
COL1A1 ACTA2 EPCAM CXCL12 CD8A KLRB1

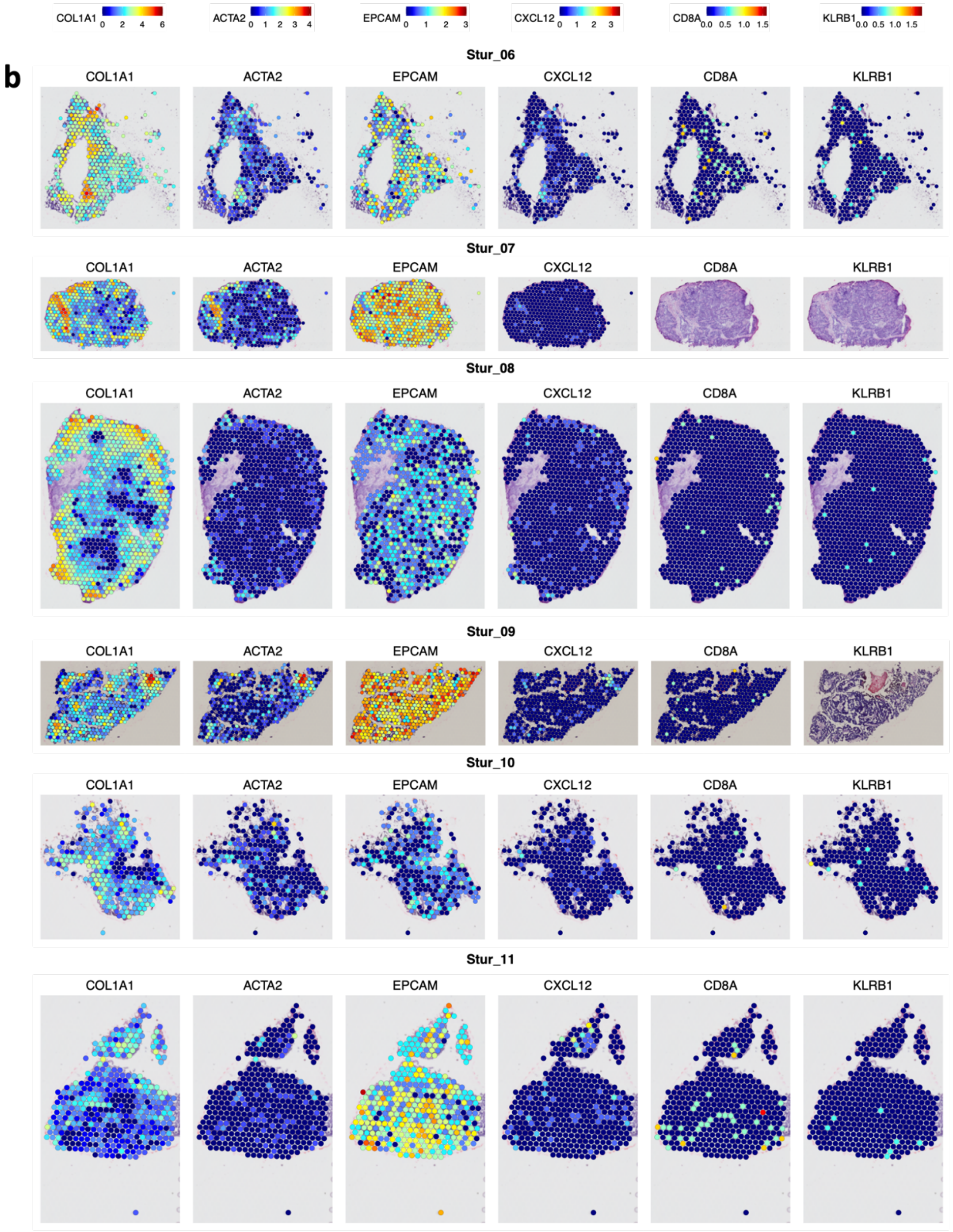

Figure S11. Spatial transcriptomics. **a** Spatial feature plots of selected marker genes of high-stromal samples and **b** low-stromal samples from the ST dataset. The samples are ordered based the average expression of COL1A1 from high (first) to low (last). Expression of the same marker across all samples shares a common color scale (tops).

Table S1. Sample and Data Information

In this study, we combined inhouse data with 3 deposited single-cell RNA sequencing (scRNA-seq) dataset. The integrated scRNA-seq dataset consists of high-grade serous ovarian carcinoma (HGSOC) samples from 20 treatment-naïve patients. We also analyzed a spatial transcriptomics (ST) dataset which consists of HGSOC samples from 11 treatment-naïve patients, and the TCGA-OV dataset (n=248).

Sample ID	Dataset Identifier and Data Type	Original Patient ID	Histology and Tumor Stage	#cells/spots used in this study
Pitt_1	Inhouse GSE232314, scRNA-seq	TP18-M419	HGSOC IIIB	7,612
Pitt_2		TP18-M509	HGSOC IVB	10,780
Regner_1	Regner et al. [1] GSE173682 scRNA-seq	Patient 8	HGSOC IIB	6,147
Regner_2		Patient 9	HGSOC IIIC	5,966
Xu_1	Xu et al. [2] GSE184880 scRNA-seq	Cancer1	HGSOC IIIB	6,421
Xu_2		Cancer2	HGSOC IIB	3,026
Xu_3		Cancer3	HGSOC IC2	3,537
Xu_4		Cancer4	HGSOC IC2	1,604
Xu_5		Cancer5	HGSOC IIB	4,422
Xu_6		Cancer6	HGSOC IIIC	3,525
Xu_7		Cancer7	HGSOC IC2	3,890
Hornburg_1	Hornburg et al. [3] EGAS00001004935 scRNA-seq	des3	Serous IIIB	7,022
Hornburg_2		des4	HGSOC IIIC	14,589
Hornburg_3		exc2	HGSOC IIIC	1,864
Hornburg_4		exc3	HGSOC IVB	3,660
Hornburg_5		inf1	HGSOC IC	3,711
Hornburg_6		inf2	HGSOC IIIC	4,823
Hornburg_7		inf3	HGSOC, unknown	1,429
Hornburg_8		inf4	HGSOC, unknown	5,618
Hornburg_9		inf5	HGSOC IIIA2	6,875
Stur_01	Stur et al. [4] GSE189843 ST data	PR_4	HGSOC, unknown	428
Stur_02		ER_6		285
Stur_03		ER_3		172
Stur_04		PR_5		170
Stur_05		ER_5		596
Stur_06		ER_4		347
Stur_07		PR_2		499
Stur_08		PR_3		949
Stur_09		ER_1		436
Stur_10		PR_1		290
Stur_11		ER_2		381

Table S2. Differentially Expressed Genes

The *FindMarkers* function from Seurat was used to identify differentially expressed genes (DEGs) between high- and low-stromal groups for each cell type. From the outputs of *FindMarkers*, we set cutoffs for DEGs.

Cell type	p_val_adj	avg_logFC
B cells, Endothelial cells	< 1e-25	Absolute value > 0.5
CA-MSCs, Fibroblasts, Myofibroblasts	< 1e-50	
$CD4^+$ T cells, Dendritic cells, Natural killer cells	< 1e-10	
$CD8^+$ T cells, Macrophages	< 1e-50	
$CD4^+$ $CD8^+$ T cells	< 1e-5	
Epithelial cancer cells	< 1e-50	

Table S3: Cytokines and Surface Proteins

Category	Gene Symbol
Cytokines [5 6 7]	AXL, BMP2, BMP4, CCL1 – CCL5, CCL7, CCL8, CCL11, CCL13 – CCL28, CCL3L1, CCL3L3, CCL4L1, CCL4L2, CCR2, CCR4, CCR5, CCR7, CCR8, CCR10, CD27, CD40, CD40LG, CD86, CSF1- CSF3, CX3CL1, CXCL1 – CXCL3, CXCL5, CXCL6, CXCL8 – CXCL14, CXCL16, CXCL17, CXCR1 – CXCR4, CXCR6, EBI3, EGFL6, EGR1, FAS, FASLG, FGF2, FGFR1 – FGFR4, FLT1, FLT3, FOXP3, GAS6, GZMA, GZMB, ICOSLG, IFNA1, IFNA2, IFNA4 – IFNA8, IFNA10, IFNA13, IFNA14, IFNA16, IFNA17, IFNA21, IFNAR1, IFNAR2, IFNB1, IFNE, IFNG, IFNGR1, IFNGR2, IFNK, IFNL1 – IFNL4, IL2 – IL7, IL9 – IL11, IL13, IL15, IL16, IL18 – IL22, IL24 – IL27, IL31 – IL34, IL37, IL1A, IL1B, IL1RN, IL2RA, IL2RB, IL2RG, IL4R, IL6R, IL6ST, IL7R, IL10RA, IL10RB, IL12A, IL12B, IL12RB1, IL12RB2, IL15RA, IL17A – IL17F, IL18R1, IL18RAP, IL21R, IL23A, IL23R, IL36A, IL36B, IL36G, ITGAV, KITLG, LAG3, LIF, LTA, LTB, MYDGF, NFKB1, NRP1, OSM, PDCD1, PDGFC, PDGFRA, PF4V1, PGF, PPBP, PRF1, TGFB1 – TGFB3, TGFBI, TGFBR1 – TGFBR3, TNF, TNFRSF1A, TNFRSF1B, TNFSF4, TNFSF8– TNFSF15, TNFSF18, TNFRSF10B, TNFSF13B, VEGFA, WT1, XCL1, XCL2
Surface Proteins [8]	ACKR1, ACVR2B, ANPEP, APP, CD163, CD274, CD44, CD93, CDH1, CDH2, CR2, EGFR, EPHA2, EPHA4, EPHB1 – EPHB4, ERBB2, FLT4, FOLH1, GNRHR, GPC3, GPR55, GRPR, IL11RA, IL13RA2, KDR, LDLR, LEPR, LRP1, MC1R, MC4R, MCAM, MET, MMP9, MRC1, MUC1, PDGFRB, PLAUR, PROM1, PTPRJ, SDC2, SELE, TACR1, TEK, VIPR1, VIPR2

Table S4: Gene-TF correlations

Within each cell type, we computed the Spearman correlation coefficient of the expression of each differentially expressed *surface protein* or *cytokine* (determined using the cutoffs in Table S2) and the activity of each transcription factor (TF, inferred via pySCENIC [9]). From the results, we set the following cutoffs to identify highly correlated gene-TF pairs. For visualization (Figures 2B, 4B, S5A, S7A), we took the union of the filtered highly correlated genes of the indicated cell types.

Cell type	To keep a gene, there is at least one TF such that	To keep a TF, there is at least one gene such that
CA-MSCs	cor > 0.4	cor > 0.5
Fibroblasts	cor > 0.4	cor > 0.7
Myofibroblasts	cor > 0.4	cor > 0.75
$CD8^+$ T cells	cor > 0.4	cor > 0.35
Natural killer cells	cor > 0.35	cor > 0.35
Dendritic cells	cor > 0.5	cor > 0.3
Macrophages	cor > 0.5	cor > 0.3

Table S5: Ligand-receptor interactions

CellPhoneDB [10] was used to identify the potential ligand-receptor for each cell type based on the raw count matrices of the high- and low-stromal groups separately. From the outputs of CellPhoneDB, we set cutoffs for ligand-receptor pairs of interest. Here "Fold change" is not a direct output of CellPhoneDB, which computes the difference of the means from the high- and low-stromal group.

Sender population	Receiver population	Means	Pvalues	Fold change
CA-MSCs	An immune population	> 0.5 in high-stromal AND > 0.5 in low-stromal	< 0.01 in high-stromal AND < 0.01 in low-stromal	> 0.1
Fibroblasts	An immune population			> 0.14
$CD8^+$ T cells	A nonimmune population	> 0.5 in high-stromal OR > 0.5 in low-stromal		> 0.32
Natural killer cells	A nonimmune population			> 0.25

SUPPLEMENTARY REFERENCES

1. Regner, M. J. *et al.* A multi-omic single-cell landscape of human gynecologic malignancies. *Mol Cell* **81**, 4924-4941 e4910, doi:10.1016/j.molcel.2021.10.013 (2021).
2. Xu, J. *et al.* Single-Cell RNA Sequencing Reveals the Tissue Architecture in Human High-Grade Serous Ovarian Cancer. *Clin Cancer Res* **28**, 3590-3602, doi:10.1158/1078-0432.CCR-22-0296 (2022).
3. Hornburg, M. *et al.* Single-cell dissection of cellular components and interactions shaping the tumor immune phenotypes in ovarian cancer. *Cancer Cell* **39**, 928-944 e926, doi:10.1016/j.ccell.2021.04.004 (2021).
4. Stur, E. *et al.* Spatially resolved transcriptomics of high-grade serous ovarian carcinoma. *iScience* **25**, 103923, doi:10.1016/j.isci.2022.103923 (2022).
5. Carrasco Pro, S. *et al.* Global landscape of mouse and human cytokine transcriptional regulation. *Nucleic Acids Res* **46**, 9321-9337, doi:10.1093/nar/gky787 (2018).
6. Zhang, Y., Guan, X. Y. & Jiang, P. Cytokine and Chemokine Signals of T-Cell Exclusion in Tumors. *Front Immunol* **11**, 594609, doi:10.3389/fimmu.2020.594609 (2020).
7. Cascio, S. *et al.* Cancer-associated MSC drive tumor immune exclusion and resistance to immunotherapy, which can be overcome by Hedgehog inhibition. *Sci Adv* **7**, eabi5790, doi:10.1126/sciadv.abi5790 (2021).
8. Ahmadi, S. *et al.* The landscape of receptor-mediated precision cancer combination therapy via a single-cell perspective. *Nat Commun* **13**, 1613, doi:10.1038/s41467-022-29154-2 (2022).
9. Van de Sande, B. *et al.* A scalable SCENIC workflow for single-cell gene regulatory network analysis. *Nat Protoc* **15**, 2247-2276, doi:10.1038/s41596-020-0336-2 (2020).
10. Efremova, M., Vento-Tormo, M., Teichmann, S. A. & Vento-Tormo, R. CellPhoneDB: inferring cell-cell communication from combined expression of multi-subunit ligand-receptor complexes. *Nat Protoc* **15**, 1484-1506, doi:10.1038/s41596-020-0292-x (2020).
