# Supplementary material for: Single-cell analysis reveals the stromal dynamics and tumor-specific characteristics in the microenvironment of ovarian cancer
Source: Commun Biol. 2024 Jan 5;7:20. doi: 10.1038/s42003-023-05733-x (PMC10770164; doi:10.1038/s42003-023-05733-x)
Supplement: Supplementary file 3 — Description of Supplementary Materials [file 42003_2023_5733_MOESM3_ESM.docx]

**Description of Additional Supplementary Files**

**File name:** Supplementary Data 1

**Description:** Source data behind the graphs in the paper
